# An Inquiry-Based Teaching Model for Nursing Professional Courses Based on Data Mining and Few-Shot Learning Technology

**DOI:** 10.1155/2022/9441375

**Published:** 2022-04-04

**Authors:** Fuling Fan

**Affiliations:** Nursing Department, Luohe Medical College, Luohe 462000, China

## Abstract

Teaching is defined as a relatively stable structural framework and activity process of teaching activities established under the guidance of specific teaching concepts and theories. It serves as a link between teaching theory and practice, as well as between the teaching system's static and dynamic conditions. The ITM (Inquiry-Based Teaching Model) has received a lot of attention and has been used in a lot of classrooms. Data mining (DM) is a method for discovering knowledge in databases and a technology for mining information in large data sets. It is primarily used to discover unknown relationships and patterns in related data. This paper applies DM's core technology, particularly the decision tree algorithm, to give hospital managers more comprehensive and in-depth data analysis capabilities, as well as strong technical support for hospitals in developing management plans. Furthermore, due to the scarcity of research data in the nursing profession, this paper introduces the few-shot learning technology to improve the model's analysis ability.

## 1. Introduction

At present, nursing students in China have low information quality, unclear information behavior, low information demand, weak learning awareness, poor literature retrieval ability, and weak information ethics awareness [1–5]. Moreover, the teaching method of nursing major is mainly classroom teaching; the teaching methods and teaching methods are relatively simple, with little contact with practice and little cultivation of students' management ability, which is not conducive to cultivating high-quality students [6]. It is an important part of nursing medicine, providing health and health services for human beings and taking the road of multicultural nursing to adapt to the changes of globalization and pluralism. Nursing education is the cornerstone of nursing career development, and how to adapt to the changes of pluralistic society is an important issue that modern nursing education should pay attention to and solve.

Data mining (DM) is an important method for discovering knowledge in databases. Data collection, processing, analysis, and interpretation are all part of the DM technology process [7, 8]. However, a study to improve the suitability and efficiency of curriculum resources is looking into how to organically combine these vast educational resources and apply them to classroom teaching according to students' interests. ITM (Inquiry Teaching Mode) emphasizes students' independent inquiry spirit, with the goal of cultivating students' innovative ability and thinking and ultimately guiding them to learn for life [9]. ITM has made significant progress in China in terms of reforming traditional teaching methods. In this case, it is decided to use DM technology to extract hidden but useful information and knowledge from these huge, incomplete, disturbing, noisy, and random data. By classifying and analyzing this potential basic information, potential problems can be identified with professional care planning and management [10, 11].

Under the guidance of modern nursing education thought, how to make full use of DM skills, integrate DM skills into subject education, and combine with the characteristics of nursing subjects to build a new education model is an important topic of education reform. In this study, DM technology is used to create a realistic scene of nursing management, provide abundant network resources, transform a single theoretical education into a rich activity experience, transform an isolated classroom space into a realistic scene of active reconstruction and closure between teachers and students, and accept open and interactive communication passively. Furthermore, because the Internet has abundant information resources and a human-computer interface that allows students to access a large amount of knowledge and information that matches their associative thinking and associative memory characteristics, students can use the Internet to generate interest in learning and encourage exploration and discovery learning [12]. Applying ITM to the course of educational research methods and exploring the innovation and practice of university teaching methods has a lot of practical implications.

## 2. Related Work

In the traditional teaching process, teachers pay more attention to the relevance, accuracy, emphasis, and difficulty of teaching content, and students are in a completely passive learning state [13]. Literature [14] puts forward the course of “inquiry learning” and points out that students' active participation in the process of scientific inquiry is the best way to learn scientific research. Literature [15] holds that science education should not require students to learn knowledge from textbooks but should master the process or method of scientific inquiry. Literature [16] holds that the content of textbooks should no longer emphasize students' passive knowledge learning but should involve them directly in the learning process, so as to cultivate students' inquiry ability and enable them to understand and master the inquiry process. According to [17], most nursing colleges do not provide multicultural nursing education, but only a few universities provide multicultural nursing education. Literature [18] holds that textbooks are an important carrier for spreading knowledge, and there is no development of education without textbooks. Therefore, it is particularly important to improve the compiling ability of multicultural nursing teaching materials and enable nursing students to acquire knowledge related to multicultural nursing in the process of nursing education. In [19], multiculturalism is regarded as a wide-ranging content, which has no clear connection and influence with the actual nursing practice. Therefore, only focus on professional knowledge education, ignoring the guidance of students' humanistic quality and cross-cultural ability. Literature [20] holds that foreign-related nursing talents should have competent humanistic professional knowledge, skilled operation skills, and nursing talents in medical and health institutions and community posts and have a high level of English to meet international nursing requirements. Literature [21] puts forward the “quality-oriented” training mode of foreign-related nursing talents in higher vocational colleges and constructs a nursing professional education program based on nursing professional ability and dominated by cross-cultural nursing ability.

In recent years, with the increasing amount of data and the increasing demand for data storage, management, and analysis, DM has gradually attracted the attention of academic and business circles. DM has been widely used in the fields of finance, commerce, and medicine to date, with positive results. Reference [22] is a rudimentary model for predicting and analyzing student dropout. It builds a university data warehouse and improves the ID3 decision tree algorithm by using educational administration and student information systems as data sources. Students' dropout can be predicted by factors such as major, percentage of failed subjects, and attendance records. The spectral clustering method is used in [23] to classify courses based on their degree of correlation, align existing courses, build an intuitive curriculum association system, and apply association rule mining to courses. Reference [24] proposed an algorithm to combine multiple subclusters based on connectivity, with the goal of improving the quality and accuracy of clustering results by focusing on the local optimality of the K-means algorithm and the sensitivity to the initial cluster centroid. Reference [25] proposes an algorithm for determining the number of clusters in a data set based on object stability. Reference [26] examines 11 widely used clustering validity evaluation indexes in depth, assessing their performance as well as the limitations of application scenarios in five aspects of the clustering algorithm.

## 3. Research Method

### 3.1. Application of DM Technology in the Nursing Field

Constructivism holds that the interaction between learners and the surrounding environment plays an important role in the understanding of learning content (that is, the construction of knowledge meaning). Under the teacher's organization and guidance, students discuss and communicate together, form a study group, analyze and discuss nursing management issues together, and conduct counseling discussion. This collaborative learning environment enables the whole group to share the ideas and wisdom of students and teachers.

With the development of nursing field, the nursing function is greatly expanded, and nursing is gradually developing towards specialization. DM technology from these huge, incomplete, noisy, fuzzy, and random data is used to discover the relationship between things through hidden but useful extracted information and knowledge to build a professional nursing quality management platform [1].

C4.5 algorithm is evolved from ID3 algorithm. An item called split information is introduced into the gain ratio. Split information is used to measure the width and balance of attribute split data. The formula is

(1) $$\begin{array}{c} SplitInfo\left(A\right)=-\sum_{i=1}^{w}\frac{s_{i}}{s}\log_{2}s_{i}. \end{array}$$

The formula for calculating the gain ratio is

(2) $$\begin{array}{c} GainRatio\left(A\right)=\frac{Gain\left(A\right)}{SplitInfo\left(A\right)}. \end{array}$$

The information entropy of classification with attribute as root node is *Entroy*(*A*), and its calculation formula is as follows:

(3) $$\begin{array}{c} Entroy\left(A\right)=\sum_{i=1}^{v}\frac{p_{i}+n_{i}}{p+n}Entroy\left(p_{i},n_{i}\right). \end{array}$$

Therefore, the formula *Gain*(*A*) for calculating the information gain divided with *A* as the root node can be obtained as follows:

(4) $$\begin{array}{c} Gain\left(A\right)=Info\left(p,n\right)-Entroy\left(A\right). \end{array}$$

After the calculation of the judgment criterion, that is, the information gain, a set of data can be obtained, which is the information gain of all attributes on the initial root node to divide the data set *S*.

The ID3 decision tree algorithm is a common decision tree learning algorithm. Its essence is to select all of the attributes of decision tree nodes based on information gain, so that all of the largest class classification nodes can have gain and the entropy of the classification data set is minimized when testing on each nonleaf. By reducing the average depth of the tree, this method effectively improves classification efficiency. To create the best decision management plan, the platform first uses a decision tree algorithm to mine and analyze these data [2]. Create a tree using this algorithm. Each node in the tree represents a single instance of a column, with the algorithm determining the node's placement, whereas each column in another instance is represented by nodes of varying depths. As a result, the algorithm is a classification form, with nodes represented as tree structures that represent additional data classification.

**Figure 1:**
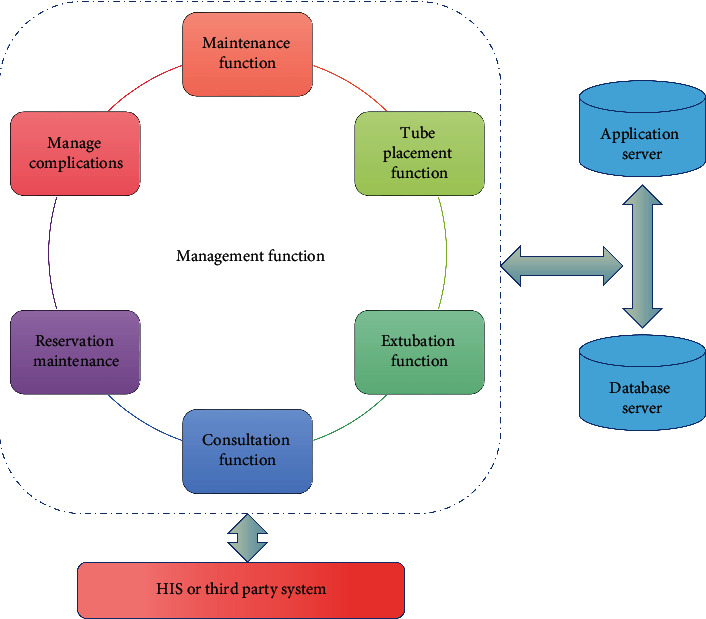
Platform architecture.

The implementation of decision tree can not only help nurses analyze the influencing factors of various clinical symptoms and implement targeted nursing actions according to the analysis results, but also effectively reduce patients' complications and improve nursing quality. It supports clinical decision-making by establishing predictive evaluation model, thus reducing medical cost and saving medical resources. Skilled nursing platform is a basic functional module, which is consistent with the clinical management of patient information through peripheral central venous catheter, including initiating clinical nurse consultation, checking professional nurse consultation table and writing consultation opinions, adding catheterization records for patients, arranging the next maintenance time, adding maintenance records, and adding complications, statistical reports, and other functions. The structure is shown in Figure 1.

Patient records in the data warehouse can be used to classify the prognosis of patients with various catheters at first and then analyze the indicators such as catheter consultation history, catheter maintenance, incidence of adverse events, etc., so as to improve related treatment schemes. In patients with catheterization, disease prognosis evaluation is an important means to promote medical quality control [6].

### 3.2. ITM Construction of Nursing Specialty Course

All teaching methods are developed by continuously verifying and fine-tuning the ideas, theoretical guidance, and teaching practice of specific teaching methods. Cognitive structuralism and constructivism are primarily at the heart of ITM's theory. The constructivist teaching theory emphasizes the fact that knowledge evolves over time. Professors cannot ignore students' prior experience because learning is active construction of knowledge based on one's own experience background. Simultaneously, the roles of teachers and students are clear: teachers serve as guides and supporters of learning, while students are active knowledge builders. As a result, constructivism theory emphasizes the importance of “situation” and “collaborative learning” in students' learning, particularly in learning environment design.

Through the mining and analysis of learning behavior data, the characteristics of students in the learning process are extracted and the learning process portrait is established. Analyze the learning time and learning quality of different students, establish a unified process description method, and make a formal description. Analyze the behavior information of students in process learning, determine the learning characteristics of the process, and set up the process portrait. Use the degree of understanding and difficulty of knowledge points embodied in learning behavior to optimize curriculum design and curriculum planning and improve existing deficiencies and improvement plans. Through the comprehensive analysis of the above two methods, data related to subject learning can be obtained, which is convenient for students to accurately get the subject learning situation and learning effect. Teachers can teach plan coordination without frequent interactive surveys and classroom tests.

**Figure 2:**
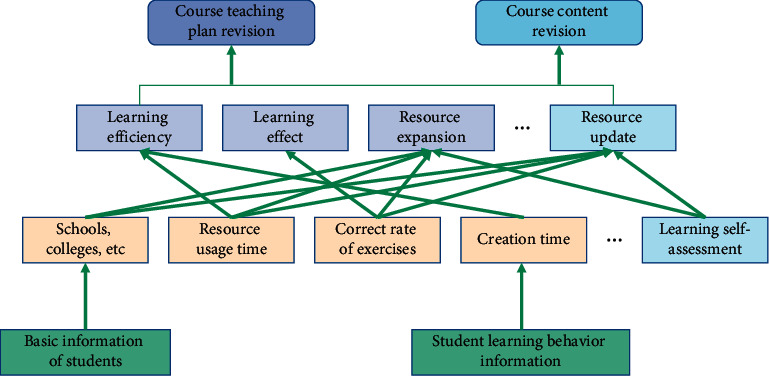
Dynamic and static data acquisition and analysis.

By summarizing, analyzing and mining the data of various learning behaviors of the course, the learning situation of course learners can be obtained to form personalized labels. This label reflects the learner's learning ability and mastery of knowledge points in the course learning. The active time for learners to explore the course content on the information education platform can reflect the demand level for the expansion and renewal of learning resources. And dynamic and static information analysis are shown in Figure 2.

Because the classroom is the main part of classroom activities, the main component of ITM is to explore the classroom through group activities. Generally speaking, the team leader should be recommended democratically, and it is better not to be appointed by the teacher. This will allow students who are responsible and supported by students to organize and lead group activities. The team leader is responsible for coordinating within the group, supervising and inspecting the team members, and contacting the teachers during the investigation. The composition of student groups can usually be determined in the first two weeks of the new school year and will not change in the next semester or even the first year. Of course, if necessary, this will change according to the specific situation, for example, after a unit or chapter is completed.

The improved K-means algorithm designed in this paper firstly defines the sample density according to the local spatial distribution of samples. You can set a density threshold to identify outliers and temporarily delete them from the data set. Then it selects an initial cluster centroid according to the density of sample distribution.

The achievement data of students in a certain course and session are represented by data set *X*={*x*_1_, *x*_2_, ⋯, *x*_*n*_}, and the data object *x*_*i*_={*i*=1,2, ⋯, *n*} in the data set is one-dimensional achievement data. *n* is the number of data points in the data set, and *k* is the number of clusters.

The distance between data points *x*_*i*_, *x*_*j*_ is

(5) $$\begin{array}{c} d\left(x_{i},x_{j}\right)=\sqrt{{\left(x_{i}-x_{j}\right)}^{2}}i=1,2,\cdots ,n;\;j=1,2,\cdots ,n. \end{array}$$

Sample distribution density of data point *x*_*i*_ is defined as the number of data points within the radius *r* around the data point:

(6) $$\begin{array}{c} de\;nsity\left(x_{i}\right)=count\left\{x|x\in X,\left|x-x_{i}\right|<r\right\}. \end{array}$$

Because there are a large number of overlapping data points in the score data, the number of overlapping data points is directly taken as the sample density value of this data point; that is, *r*=1 is taken.

The threshold value of sample density is determined by multiplying the average value of sample density of all data points by a coefficient *c*:

(7) $$\begin{array}{c} threshol\;d=c\times \frac{1}{n}\sum_{i=1}^{n}density\left(x_{i}\right). \end{array}$$

Pay attention to cultivating students' innovative spirit and practical ability through examination, cultivating good psychological quality, learning interest and positive emotional experience, respecting individual differences, recognizing the uniqueness of individual development, giving positive evaluation, and giving full play to students' multiple potentials [20].

Teachers play an important role in assisting and guiding students in inquiry learning, which is centered on students' self-exploration process. Teachers should assist and guide students as needed throughout the learning process and fully reflect their leadership abilities. Students' time and homework must be carefully planned by teachers. Inquiry learning in the context of an inquiry-based teaching management platform relieves teachers' workload while also testing their professional abilities. Teachers' primary role in inquiry-based classroom instruction is to guide and inspire students, as well as to improve their learning and problem solving abilities. In a timely manner, summarize and evaluate the discussion and communication process, as well as the outcomes of the discussion and communication. Teachers and students collaborate in inquiry classroom teaching. They are clearly teachers and masters when they enter the inquiry classroom [13].

## 4. Results Analysis and Discussion

### 4.1. ITM Application Analysis

All kinds of teaching methods have relatively fixed teaching methods, and the active exploratory learning course is not static, but flexible and diverse. Let students study and discuss problems through independent thinking, group discussion and group communication, find solutions to problems, acquire relevant knowledge and resources, and combine and summarize classroom teaching applications and student feedback. Through the teaching system, abundant knowledge and information resources are provided, so that teachers and students can easily obtain the required information from the system, and at the same time, the natural and convenient navigation system can be exchanged to enable students to quickly complete self-study activities. In this way, teachers can avoid spending a lot of time and energy to master deep network computer knowledge and making educational software, so that teachers can concentrate on educational design and students will not get lost and struggle, in addition to the influence of bad information.

**Figure 3:**
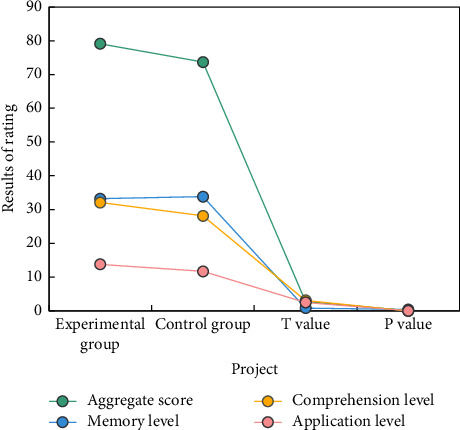
Examination results of nursing management knowledge and skills of nursing students.

QQ chart and QQ residual were used to test the normality of evaluation results of nursing management knowledge and skills of nursing students. From QQ chart and QQ residual chart, the evaluation scores of nursing students obey normal distribution.  Figure 3 shows the test results of the evaluation results of nursing management knowledge and skills of two groups of nursing students.

According to the evaluation scores of EG (experimental group) and CG (control group) nursing management knowledge and skills, as well as statistical tests, the total score, comprehension level, and application analysis level of EG nursing students, are higher than those of CG nursing students, with no significant difference in memory scores. Inquiry-based learning is based on the principle of interaction between two subjects, emphasizing learners' active participation, teachers' proper guidance, and students' active participation in the learning process. A learning method for gathering, organizing, and actively mining knowledge is included in the materials. Students can develop not only scientific knowledge, but also scientific thinking and attitude in this way. In general, teachers incorporate knowledge and skills into problems, allow students to learn knowledge while solving problems, and use knowledge to improve skills, all of which exemplify the concept of “hands-on learning.”

**Figure 4:**
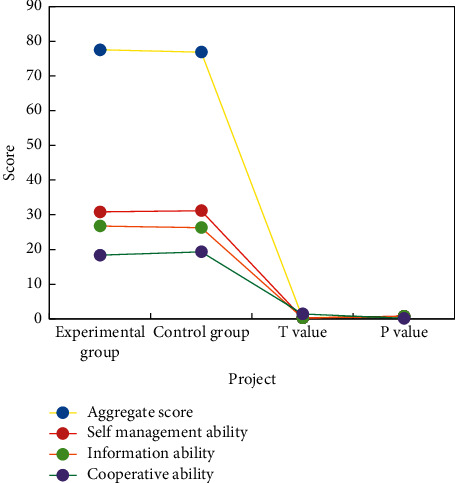
Comparison results of total scores and scores of all dimensions of autonomous learning ability of nursing students (before intervention).

Figure 4 shows the comparison between the total score of self-study ability before experimental teaching (before intervention) and scores of various dimensions of EG and CG nursing students.

According to the statistical test, there was no significant difference between the scores of self-study ability of EG nursing students and CG nursing students before the intervention. Step-by-step explanation of new knowledge points put forward by students aims at combining and discussing new knowledge points and practical teaching methods with students, instead of completely brainwashing and gradually injecting new knowledge into students' learning process. The ultimate goal of group exploration is to complete the general tasks stipulated in this chapter and require students to plan and design in various aspects after mastering the basic knowledge. They must perform a set of specific steps, such as division of labor, time planning, provision of materials, data collection, running experiments, and writing reports. The students' ability to accomplish the activity objectives is evaluated by concrete demonstration and project description.

**Figure 5:**
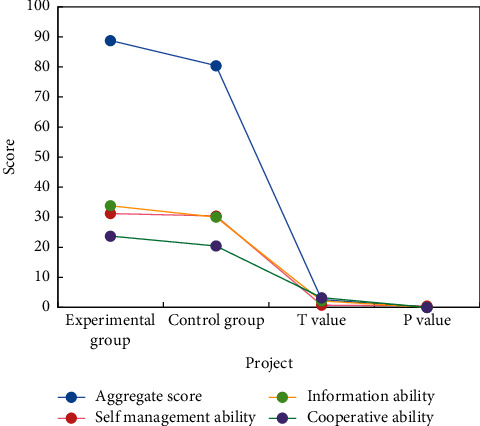
Comparison results of total scores and scores of each factor of independent learning ability of nursing students (after intervention).

After the intervention, the scores of self-learning ability, information ability, and cooperation ability between EG nursing students and CG nursing students were significantly different, but there was no significant difference in the scores of self-management ability (see Figure 5).

Nursing is a discipline with strong theoretical knowledge, which contains many concepts, principles, and abstract theories, which makes students feel bored. The research shows that most CG students who adopt traditional teaching methods lack interest and enthusiasm in nursing management. ITM of nursing specialty courses based on DM skills has significantly increased students' interest in learning, enhanced their learning enthusiasm and initiative, and increased the fun and intuition of education. This stimulates students' interest in learning and makes learning more active. Based on the course ITM of DM skill nursing specialty, this study makes full use of DM skill simulation to create a realistic nursing management scene, and students use the knowledge they have learned to conduct nursing management activities in the simulation situation, thus transforming into a single theoretical teaching, with rich experience in activities in class. Therefore, the nursing professional course ITM based on DM skills can improve students' problem analysis ability and practical problem solving ability and help to cultivate nursing management skills.

**Figure 6:**
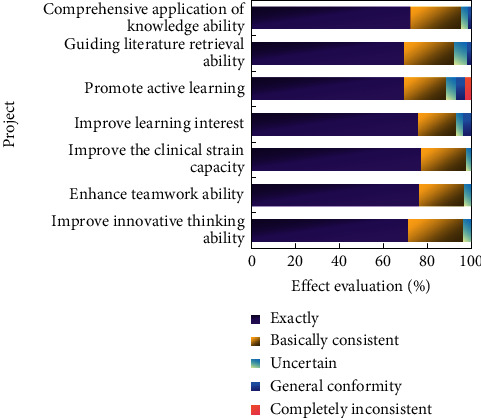
EG students' evaluation of the effect of inquiry-based teaching.

The network-based education has clear learning objectives and content, as well as related tasks and problem situations. Students are primarily responsible for learning objectives throughout the educational process, aside from the appropriate inspiration, guidance, and assistance from teachers. It conducts independent exploratory learning in a variety of ways, actively refers to and collects reasonable data, combines and processes it, and finally achieves the goal of solving problems and acquiring knowledge and ability, all while being guided and driven by problems or tasks. Students have a lot of freedom to study independently with this multichannel problem solving method. As a result, an ITM-based nursing professional course based on DM technology can help students improve their autonomy and discipline awareness. Consider internal medicine, which is at the heart of the nursing process. In terms of the knowledge system and curriculum requirements, ITM does not contradict nursing education [4, 5]. This model integrates the cultivation of innovative ability, clinical thinking ability, and cooperation ability into nursing education in the new era, trains innovative applied nursing talents with innovative spirit and adapting to the development of the times, promotes nursing education's innovative development, and thus produces better teaching effect for nursing students (Figure 6).

From these analysis results, the comprehensive inquiry-based teaching concept can improve the innovative thinking of undergraduates. In the interview, students thought that inquiry education provided a platform for innovation, guided scientific research, and helped innovation. This is basically consistent with many scholars' research on providing an innovation platform for college students to improve their innovation ability [6, 7].

**Figure 7:**
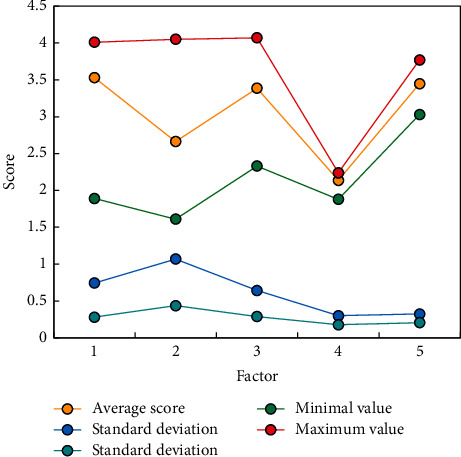
Scores of various factors in cross-cultural sensitivity scale.

The concept of inquiry teaching supporting nursing practice education puts forward a higher level of professional test for teachers. Teachers should organize and carefully consider all aspects of educational activities. In the whole process of maker education, teachers should learn to integrate and help students innovate and share their successful creation. This will greatly develop and improve teachers' abilities and improve the quality of comprehensive education for nursing teachers. In order to analyze the poor cross-cultural sensitivity of the interviewees in more detail, the multicultural sensitivity was counted according to the factors shown. See Figure 7 for details. Among them, 1∼5, respectively, represent communication participation, difference of identity, communication confidence, communication pleasure, and communication concentration.

The results show that the scores of each factor from high to low are communication participation, communication concentration, communication trust, discriminatory identity, and communication pleasure. Among them, the scores of communication participation and communication focus are relatively separated, indicating that modern nursing students are willing and able to engage in cross-cultural communication seriously. But differences in identity and communicative enjoyment were relatively poor, suggesting that, despite cross-cultural communication, students were less able to distinguish between cultural differences due to a lack of understanding of other cultural differences, affecting the pleasure of communication. Therefore, it is suggested that follow-up research work should focus on cross-cultural knowledge learning.

### 4.2. DM Analysis

The distribution of scoring data has the following characteristics: First, the distribution of scores in the same subject may fluctuate greatly due to changes in subject content, question types, examination difficulty, and so on. Secondly, in the case of different subjects in the same academic year, the distribution of scores may be quite different due to the great differences in subject contents and examination methods. Third, the scores of most subjects are distributed continuously, without obvious flaws, and the number of intermediate students is large. Fourth, the scores of individual students in most subjects are not good, so they are separated from the scores of most students and become outliers in the data set. Calculate the standard deviation of sample size and contour coefficient for the discretization results of the three algorithms.

**Figure 8:**
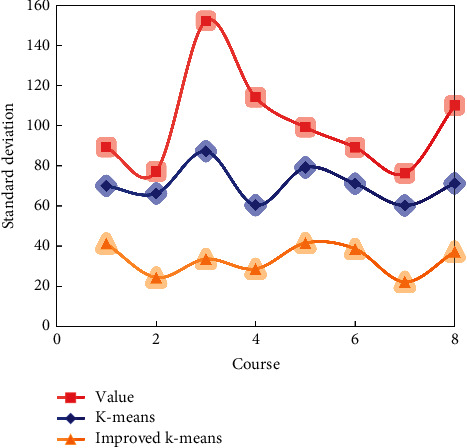
Standard deviation of the sample number of results of data discretization.

Figure 8 shows the standard deviation of the result sample size of three methods of discretizing the result data of some courses according to the score value by using the K-means algorithm and the improved K-means algorithm.

**Figure 9:**
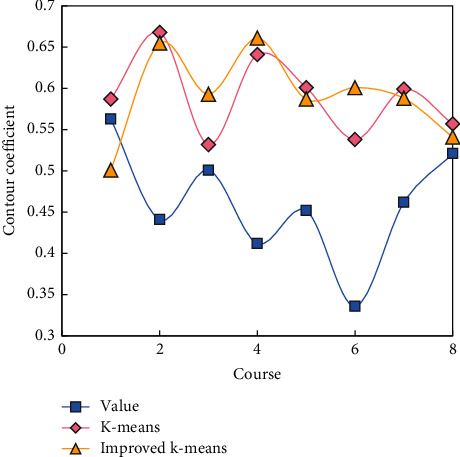
Contour coefficient of result of data discretization.

As can be seen in Figure 9, the result of fractional discretization is not good in data similarity and does not meet the requirement of discretization, so as to maximize the data similarity within clusters and the data difference between clusters.

The improved K-means algorithm of data discretization can make the data similarity within segments and data differences between segments as large as possible and make the number of data points more uniform, meeting the requirements of class discretization.

It is worth noting that ITM injects a lot of color into teaching and teacher evaluation methods, as well as raising the bar for teachers, students, the educational environment, and educational conditions. Teachers' knowledge scope, classroom control, students' understanding, and instructional design skills are all put to the test in ITM. And teaching methods are not set in stone. Teachers must pay attention to course effectiveness, integrate various teaching methods based on the actual teaching situation in the classroom, and fully exploit the benefits of each teaching method. ITM, on the other hand, is time-consuming and labor-intensive, making it unsuitable for classes with a large number of students. As a result, teachers should adapt it to the situation at hand. Dynamic data differs from static data in that it can be changed. Bring course learners' learning habits into the information-based teaching platform. Compare the time spent browsing course content, the correct rate of answering course matching questions, and the knowledge point feedback scores. These data reflect students' interest in and mastery of the course, and self-tests and other tests can be used to achieve the online learning effect. The learner's self-evaluation of the mastery degree of the learning content, such as complete mastery, basic mastery, and unclear places, is referred to as a self-test. It comes with knowledge test questions that the system evaluates based on the answers to the test questions.

The ITM course of nursing specialty constructed by researchers has achieved relatively successful results. In the inquiry teaching of nursing major courses, researchers guide students to learn independently, adopt flexible and diverse teaching methods around the teaching content, and make the teaching content more colorful, thus stimulating students' learning subjectivity and learning better and constantly deepening students' understanding of multicultural nursing, so that students can enhance their memory and absorption of multicultural nursing knowledge through multiangle and novel ways. According to the evaluation results of teaching methods, students have a high degree of recognition of the teaching methods and means adopted in this study, which has produced positive and effective results.

## 5. Conclusion

In the era when DM realizes personalized learning with big data, big data is influencing and changing the ecological environment of education and the operation mode of teaching. Using various related technologies of DM to deeply process the massive data of the platform can make the information system get rid of the previous mode which can only be used as electronic data entry tool, simple query function, and statistical report. Evaluation feedback in each teaching stage is used to continuously improve and perfect teaching activities, promote students to better understand and apply DM knowledge and concepts, and improve the quality of engineering education. Under DM technology, the nursing professional course ITM has changed the teaching concept, established a brand-new modern nursing education concept, effectively cultivated the nursing professional students' autonomous learning ability, auxiliary learning ability, and practical problem solving ability, laid a solid foundation for the full implementation of student-centered quality education, and provided reference for the development of inquiry teaching.

## Data Availability

The data used to support the findings of this study are included within the article.
